# Development of a stroke network in Kinshasa (Democratic Republic of the Congo): a humanitarian project of the Italian Stroke Association—Associazione Italiana Ictus

**DOI:** 10.3389/fstro.2024.1305123

**Published:** 2024-04-29

**Authors:** Lorenzo Benedetti, Chadrack Bosenedje, Anne Falcou, Jonathan Mabiala, René Lumu, Antonio Ciacciarelli, Rémy Kashala, Danilo Toni

**Affiliations:** ^1^Department of Human Neuroscience, “La Sapienza” University of Rome, Rome, Italy; ^2^Monkole Hospital, Kinshasa, Democratic Republic of Congo; ^3^Emergency Department Stroke Unit, Hospital Policlinico Umberto I, Rome, Italy

**Keywords:** stroke, stroke unit, low-middle income countries, Democratic Republic of the Congo, project, humanitarian

## Abstract

In sub-Saharan Africa (SSA), stroke incidence is ~316 cases per 100,000 inhabitants and in 75% of SSA countries stroke is among the top three causes of death. This epidemiological evolution results from the exponential growth of the African population as well as the lack of policies for prevention and therapy. The Democratic Republic of Congo is one of the countries included as a “low-income country” in the 2023 Organization for Economic Co-operation and Development's Development Assistance Committee List; its capital, Kinshasa, is a megalopolis with more than 18 million inhabitants. According to our knowledge, no hospital in Kinshasa is currently capable of admitting and treating patients with stroke according to international guidelines. The Italian Stroke Association—Associazione Italiana Ictus (ISA-AII) is a multidisciplinary scientific society for the fight against cerebrovascular diseases. Some members of the ISA-AII joined to form a working group dedicated to the care of stroke in Africa. In this article, we explain the project designed by ISA Africa that, in collaboration with the referral hospital of a district of Kinshasa, has the aim of building a network for diagnosing and treating stroke, including ambulance services, a stroke team in the emergency room, a stroke unit, an outpatient clinic, and primary prevention activities among the population.

## 1 Rationale

In the past 20 years, there have been significant improvements in the diagnosis and management of stroke, leading to a decrease in related morbidity and mortality almost worldwide. This decrease is mainly due to the diffusion of stroke units (SUs) and the efficacy of recanalization procedures for ischemic stroke. In fact, SUs have consistently proven to increase the probability of survival, returning home, and regaining complete autonomy in daily life after a stroke, regardless of clinical severity, sex, or type of stroke, either ischemic or hemorrhagic (Stroke Unit Trialists' Collaboration, 2013).

Unfortunately, these advancements are not yet available in sub-Saharan Africa (SSA), where the incidence of stroke is still rising (Thayabaranathan et al., 2022) and stroke mortality rate is up to five times higher compared to high-income countries (HICs) (Leone et al., 2021). In low- and middle-income countries (LMICs) situated in SSA, the prevalence of stroke ranges from 15 to 1,460 per 100,000 people, while its incidence is estimated to be between 25 and 250 per 100,000 people per year (Adoukonou et al., 2021). The Global Burden of Disease Study 2019 estimates that the age-standardized incidence of stroke from 1990 to 2019 increased by 1.89% in southern SSA (Feigin et al., 2021).

The surveillance of vascular risk factors in SSA over the past decade indicates that most adults are exposed to at least one modifiable risk factor, such as hypertension, diabetes, cigarette smoking, alcohol abuse, unhealthy diet, physical inactivity, obesity, or hypercholesterolemia (WHO, 2015). The main causes and risk factors of stroke in SSA differ from those observed in high-income countries (HICs), in which non-modifiable risk factors play an important role: In SSA, it has been estimated that modifiable risk factors account for more than 80% of stroke risk (O'Donnell et al., 2010). The major modifiable risk factor for stroke in SSA is arterial hypertension (Akinyemi et al., 2021), which affects between 16 and 40% of the adult population. Despite these significant data, most people are not aware of their possible condition (Ataklte et al., 2015; Geldsetzer et al., 2019). Human immunodeficiency virus (HIV) infection is considered the second main risk factor, but for people younger than 45 years, it is the main risk factor (Akinyemi et al., 2021)^1^. Another important cause of stroke in SSA is sick cell anemia, which is characterized by a bimodal age distribution: most strokes occur before the age of 20 years and after 30 years, with a peak incidence at ages 10–15 years (Akinyemi et al., 2021).

The Democratic Republic of Congo (DRC), an SSA country, is one of the countries listed as a low-income country in the 2023 Organization for Economic Co-operation and Development Assistance Committee List. There are few studies about the epidemiology of stroke in the DRC, but a systematic review highlighted that, in SSA, the DRC has the biggest incidence increase over the last decades, with an incidence rate of +27.8% between 1990 and 2010 (Owolabi et al., 2015). The age-adjusted death rate is 120.4 per 100,000 people, and stroke-related deaths have reached 6.9% of total deaths in the country, according to World Health Organization data for 2020^2^. The joint mission of the United Nations Inter-Agency Task Force on the Prevention and Control of Non-Communicable Diseases visited Kinshasa in 2015; the main conclusion was to increase advocacy for including chronic diseases, such as cardiovascular and cerebrovascular diseases, on the agenda of key government priorities in the DRC^3^. The objective of this joint UN interagency mission was to initiate a coherent and coordinated approach for developing a multi-sectoral national action plan for controlling non-communicable diseases (NCDs) while setting realistic national targets and priority actions to be taken between 2015 and 2025 to reduce premature mortality associated with NCDs. However, to our knowledge, no concrete action has been taken yet.

Currently, the only stroke therapy that appears readily applicable in LMICs is the creation of SUs. Indeed, recanalization therapies are not yet considered reasonably suitable because of the shortage and cost of thrombolytic drugs, the absence of angiography rooms, and the lack of physicians' skills. Langhorne et al. evaluated SU applicability in such settings (Langhorne et al., 2012). They used four items to address this issue: (1) the potential worldwide effect of this care, (2) the available evidence for implementing SUs outside HICs' healthcare systems, (3) the components likely to be key in LIMCs' SUs, and (4) how to overcome the barriers to implementing SUs in LMICs. Regarding the first item, they found two studies (Gilligan et al., 2005; Langhorne et al., 2009) that demonstrated how SUs are the most effective population intervention worldwide, at least in terms of independent survival. Concerning the second point, they found several studies from five continents that, despite using different methodological approaches, highlighted lower mortality rates in the LMIC SU group compared to control groups (patients not hospitalized in an SU) and that the difference was always statistically significant. Analyzing the possible key components in LMICs' SUs, they discovered that structural elements, such as easy access to diagnostic imaging and supporting investigation, could represent an obstacle. On the contrary, multidisciplinary teams, acute management and monitoring, and early rehabilitation programs could be fundamentals. Finally, one way to overcome the barriers to implementing SUs in LMICs could be to create a stroke path at the national, regional, or local level and set up a geographically defined hospital area with medical supervision, skilled staff, and basic equipment and medication. The results need to be interpreted because most of the reviewed studies were conducted in big cities without considering the realities of rural areas. Despite the likely lack of resources in the setting under analysis, this information remains important given the evidence of the statistically significant effectiveness of SUs. Nonetheless, a recent report by the World Stroke Organization noted highly inadequate territorial coverage of neurovascular units in LMICs, with only 18% having adequate coverage compared to 91% of HICs (*p* < 0.001), as well as an almost total absence of cerebrovascular disease prevention actions in SSA (Owolabi et al., 2021).

## 2 Objective

The main objective of our project is to build a network for stroke prevention and treatment in a region of the world where, at the moment, there is no structure for doing so according to international recommendations.

## 3 Material and methods

The Italian Stroke Association—Associazione Italiana Ictus (ISA-AII) is a multidisciplinary Italian scientific society whose mission is to fight and prevent stroke and other cerebrovascular diseases. Owing to the initiative of some ISA-AII members in 2023, ISA Africa, a working group currently made up of 70 people, including doctors, nurses, and specialists in stroke care, was created. The aim is to combine forces and skills to promote stroke culture and care in SSA countries. The main objectives are

- the implementation of training courses on stroke for African healthcare workers,
- the organization of congresses and conferences on stroke dedicated to students and specialists from SSA countries,
- the creation of protocolled pathways for stroke care (including SUs and telemedicine),
- the organization of fellowships (economically supported) of healthcare workers from SSA in Italian SUs, neuroradiology, and neurorehabilitation services, and
- the promotion of clinical research on cerebrovascular diseases in SSA.

During previous humanitarian missions in Kinshasa, the capital of the DRC, we observed that neither the public university hospital (Unikin) nor other public and private hospitals had a dedicated stroke management system. Consequently, ISA Africa established a collaboration agreement with the Centre Hospitalier of Monkole (CHM), the district's referral hospital for Mont-Ngafula municipality in Kinshasa. Mont-Ngafula is a district of Kinshasa with 261,000 inhabitants for an area of 358.92 km^2^. CHM opened on 26 March 1991, with a ministerial authorization from the Ministry of Health dated 15/09/1990 n° CAB/MIN/SP/1837/90. Born from the impulse from a group of Congolese and Europeans residing in Kinshasa within the non-profit Centre Congolais de Culture, de Formation et de Développement, the hospital provides medical, pediatric, and surgical emergency services, which are open 24/7, and it has radiology services and a 16-slice computed tomography scanner. It also has departments for internal medicine, gynecology, pediatrics, and surgery and an intensive care unit. The internal medicine department includes 19 beds and exploits the collaboration of specialists in endocrinology, cardiology, pulmonary medicine, and gastroenterology. In 2022, 167 patients were hospitalized for stroke at the CHM: 71 in internal medicine and 96 in intensive care wards. With an estimated stroke incidence of ~316 cases per 100,000 inhabitants per year (Akinyemi et al., 2021) and considering that Mont-Ngafula has ~261,000 inhabitants, the number of stroke patients is significantly lower than expected, which means that most stroke patients do not even have access to hospitalization and, consequently, to appropriate treatment. Most stroke victims are likely to stay at home and not access the necessary treatment in a dedicated facility, despite demonstrations that such facilities are effective and efficient in improving the prognosis of stroke.

**Figure 1:**
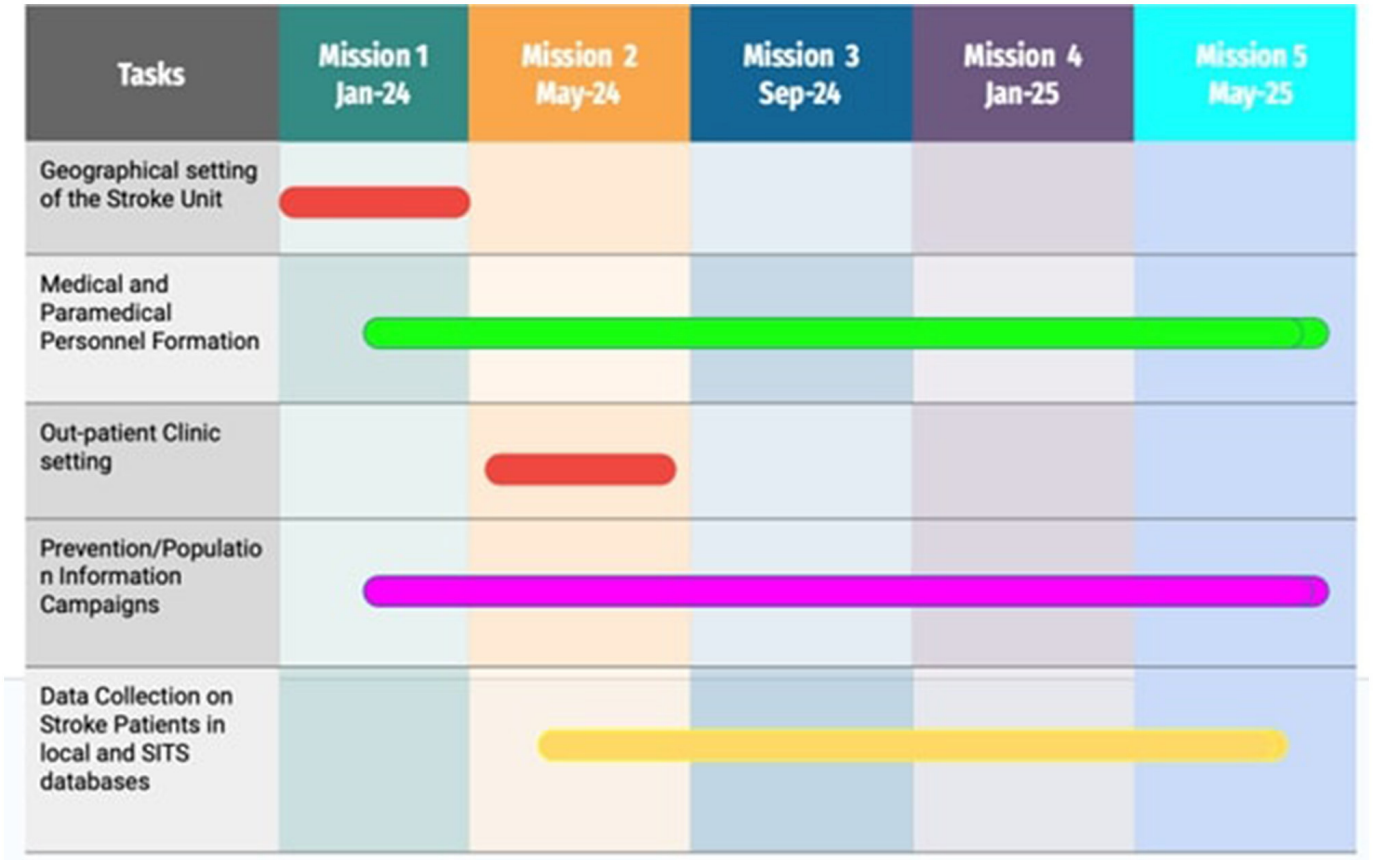
Milestones of the project.

Our project is based on a series of field missions, 5 in 18 months (Figure 1), with the following aims:

1. determining the geographical location of the SU inside the hospital and finding the material needed to set up the SU (aimed to be opened in January 2024);

2. training staff, doctors, and nurses; and

3. gradually creating a stroke network, based on

• **territorial medical assistance** by setting up a dedicated telephone number. The call activates an ambulance with a doctor on board, who is trained to provide first aid and identify the signs and symptoms of suspected stroke (application of an early diagnosis scale as the Face, Arm, Speech and Time scale (FAST) or the Cincinnati Pre-Hospital Stroke Scale (CPSS)). In the case of a suspected stroke, the patient will be associated with a Stroke Code and will be immediately transported to the →

• **CHM emergency room**, where they will be assisted by an expert stroke team (medical and nursing staff) that will manage the stroke as best and as quickly as possible according to the Stroke Diagnostic-Therapeutic Assistance Pathway. The doctor will conduct a detailed medical history to determine the time of onset of symptoms and/or the time at which the patient was last seen in good health and a neurological examination to establish the Neurological Institute of Health Stroke Scale (NIHSS) score, which quantifies the extent of neurological deficit. At the same time, nurses undress the patient, insert a venous access and bladder catheter, check vital signs, record an electrocardiogram, and begin continuous monitoring of cardiac activity. At the end of this first classification, the patient will be taken to the radiology unit as fast as possible to carry out a →

• **cerebral computed tomography without a contrast agent** to distinguish between ischemic and hemorrhagic strokes.

• **the SU**, where, once the emergency department phase, overall patients suffering from ischemic or hemorrhagic stroke in the acute or subacute phase will be admitted to undergo

○ treatment for the acute event following therapeutic protocols in line with international recommendations;
○ nursing to prevent stroke complications (infections, bedsores, heart rhythm disorders, myocardial infection, etc.) and ensure correct hygiene, hydration, and nutrition;
○ early high-intensity rehabilitation (early passive mobilization in bed, occupational therapy, and early out-of-bed mobilization, including sitting, standing, and walking), and speech therapy;
○ complete etiological diagnostic workup to establish the cause of the stroke (ultrasound examination of the supra-aortic arteries, transthoracic echocardiogram, continuous recording of cardiac activity, lipid profile, HIV serology, etc.) and, therefore, plan the best secondary prevention therapy (antiplatelets, anticoagulants, statins, carotid surgery, etc.).

4. Opening of a neurovascular outpatient clinic (aimed to be operative by the end of May 2024), dedicated to primary prevention for people with cardiovascular risk factors and the follow-up of the discharged patients from the SU (systematic outpatient visits at 1, 3, 6, and 12 months after discharge).

5. Organizing stroke prevention activities using information campaigns on television and radio (once a month for 18 months) and screening days in schools and parishes (two in every mission). All those found to be at risk will be referred to the CHM neurovascular clinic.

Once this network has been built, the plan is to make CHM fully autonomous in the management of stroke while maintaining permanent collaboration relationships for professional updates, collegial discussions of complex clinical cases, and the exchange of resident students.

## 4 Data analysis

The following demographic, clinical, and follow-up data will be recorded in a database:

- ID number
- Date of birth
- Race
- Gender
- Ischemic/hemorrhagic stroke
- NIHSS at baseline
- NIHSS at 24 h, 7 days, discharge, 3 months
- Modified Rankin Scale at 3 months
- Antiplatelets/anticoagulants
- Lipid-lowering treatments
- Antihypertensive treatment
- Adverse events during hospitalization
- Length of stay
- Discharge destination

We will analyse data collected in the database every 6 months (May and December 2024) and at the end of the project (May 2025).

The efficacy and effectiveness of the system will be assessed using the following criteria:

- The number of emergency interventions in the territory
- The number of patients admitted to the emergency room with a stroke code
- The number of patients hospitalized in the SU
- The outcome at discharge: mortality and disability (modified Rankin Scale)
- The number of patients followed at the outpatient clinic in 12 months and, consequently, the number of patients lost in the follow-up

The criteria outcomes at discharge will be compared with those obtained during 2023. Other criteria are not comparable to those in previous years when the stroke network did not exist.

Parametric and non-parametric tests will be used depending on the distribution of the variables, and the analysis will be conducted using SPSS (version 27). The basic characteristics and follow-up results will be presented for all patients. Categorical data will be reported as frequencies and proportions, while continuous data will be presented as means and standard deviations or medians and ranges, depending on the case. Comparison between groups, before and after the data collection period, will be done using a chi-square or Fisher's exact test for categorical variables and an analysis of variation or Student's *t*-test (or Mann–Whitney *U*-test) for continuous variables. The significance level will be set at a *p*-value of < 0.05.

## 5 Conclusion

We are optimistic that this is just the beginning of a series of long-term projects; future projects could include

- bringing in other national and international scientific societies to broaden the scope of interventions;
- creating a supply pathway for thrombolytic drugs, enabling local residents to receive free pharmacological reperfusion; and
- creating large-scale local initiatives in which trained African health workers could expand the training chain to areas that are hard to reach through our limited missions.

Filling the gap between the indications of international guidelines and the current state of the art in the DRC will be a long road. We believe that our missions will lay the foundations for a solid and fruitful collaboration that can contribute to overcoming a great health inequity by providing populations in LMICs with treatments like those available in HICs.

## Data availability statement

The original contributions presented in the study are included in the article/supplementary material, further inquiries can be directed to the corresponding author.

## Author contributions

LB: Writing – original draft, Writing – review & editing. CB: Writing – original draft. AF: Writing – original draft, Writing – review & editing. JM: Supervision, Writing – review & editing. RL: Writing – original draft. AC: Data curation, Formal analysis, Writing – review & editing. RK: Writing – review & editing. DT: Writing – original draft.

## Footnotes

^1^ https://ourworldindata.org/life-expectancy

^2^ https://data.who.int/countries/180

^3^ https://www.who.int/publications/i/item/WHO-NMH-NMA-17.64
